# Supplementary material for: Patient–Practitioner–Environment Synchronization: Four-Step Process for Integrating Interprofessional and Distinctive Competencies in Osteopathic Practice—A Scoping Review with Integrative Hypothesis
Source: Healthcare (Basel). 2025 Apr 4;13(7):820. doi: 10.3390/healthcare13070820 (PMC11989069; doi:10.3390/healthcare13070820)
Supplement: Supplementary file 1 [file healthcare-13-00820-s001.zip › Table S1.pdf]

healthcare

MDPI

**Table S1. Included articles, relevant contents, emergent themes, and additional references.**

| Included article | Relevant contents | Emergent subthemes | Additional References |
|---|---|---|---|
| Accardi et al., 2023 [56] | *"In the context of osteopathic care[...]It also relies on the practitioner's ability to perceive the intended meaning behind the child's gestures, postures, movements, and expressions, as well as the timing and emotional attunement during reciprocal embodied communication. In clinical encounters involving caregivers, children, and parents, individuals' lived bodies are mutually coupled through eye contact, gaze, facial expressions, voice, gestures, positioning, movements, and intentional actions"* | Narrative-based sense-making and decision-making process | Liem and Neuhuber 2020 [74]; Lunghi et al., 2021 [75]; |
| | *"Therapeutic touch has the potential to elicit an affective response and influence the body schema/perception[...] Therapeutic interventions, guided by an enactivist perspective, facilitate insight into patients' feelings and dominant patterns of sense-making "* | Touch-based shared sense-making and decision-making process | |
| | *The ability of osteopaths to utilize affective touch, mediated by CT fibers, in initial interactions activates these fibers, leading to autonomic, neurochemical, and behavioral responses that may enhance the child's receptivity to osteopathic manipulative treatment […]in which the practitioner and child synchronize bodily actions and interactions, co-creating meaning and coordination. […]. Dynamic stroking […] activates CT fibers and the posterior insular cortex in both mothers and infants, promoting the development of regulation, synchronization, and anticipation of sensory stimuli.* | Hands-on, mindfulness-based OMT | |
| | *"Osteopaths can involve parents in their child's health process, highlighting their parenting skills and teaching them reproducible techniques for home use, such as gentle touch and skin-to-skin contact. This approach facilitates a holistic adjustment for both parent and child."* | Osteopathic active-participative approaches | |
| Arcuri et al., 2022 [57] | *[...]theoretical models such as the biopsychosocial model (i.e. effective communication to improve therapeutic alliance), and to complexity medicine framework (i.e., Cinefyn to address decision-making processes) (P4, P5, P9) were among the proposed strategies."* | Narrative-based sense-making and decision-making process | Lunghi et al., 2021[75]; Lunghi and Liem 2020 [10]; Liem and Lunghi 2023 [76] |
| | *"Participants showed heterogeneity in the management of clinical uncertainty. It has been reported that somatic dysfunction (SD) was used as a simplifying tool for decision-making (P1, P3);.[...] SD as a safe tissue-touch based communication tool between operator and person complex adaptive health system.[...]In other terms, it is evaluated in a shared decision-making process if a specific type of touch, executed in a particular area, can evoke perceived changes in patient agency, in the ability to perform daily movements, or perform a specific objective examination test. During the assessment of SD, an area of interest for both patient and osteopath, practitioners investigate patients' body awareness to better understand their psychological perceptions in exploratory verbal and non-verbal communication."* | Touch-based shared sense-making and decision-making process | |
| | *"OMT application to distal body areas apparently not connected with patient complaints, but perceived as linked with the symptomatic region by the patient, could be seen as a body awareness improvement strategy."* | Hands-on, mindfulness-based OMT | |
| | *"[...]participants reported the importance of making the patient active and participating, through sharing the therapeutic approach and using enactive strategies to improve body awareness. As it was reported by P5 the patients are involved to improve their agency: "It is necessary to make the patients understand that something is changing by looking at his daily life habits: how he walks, how he stands, how he washes, how he dresses, how his work is going or how better he can work."* | Osteopathic active-participative approaches | |
| Arrigoni et al., 2024 [34] | *"Successful interpersonal interactions develop reciprocal cycles of action and perception adapt neural pathways, enhancing predictability and reducing uncertainty through shared narratives"* | Narrative-based sense-making and decision-making process | McParlin et al., 2022a [29]; McParlin et al., 2022b [77]; |

| | | | |
|---|---|---|---|
| | *"[...]Participants refer to SD as an outcome of a touch-based participative interactive process between osteopath and patient, also shared by verbal communication respectful of the patients' expectations. [...]The Enactive-Neuroaesthetic model aids in understanding patient responses during sessions. [...]Emphasizing touch and seeking patient feedback are also pivotal.[...]This perspective can help clinicians understand conditions that patients may not want to or cannot express in words. By evaluating and enhancing signals concerning the perceptual and motor systems of patients, clinicians can grasp these unspoken conditions. By synchronising reciprocal gestures and sharing bodily cues, clinicians foster positive therapeutic relationships."* | Touch-based shared sense-making and decision-making process | |
| | *"Therapeutic touch [...] is seen as a powerful tool for building alliance and synchrony in the context of manual therapy, […] to recognize individual preferences and sensitivities and to capture patient feedback through spontaneous expressions and movements, which indicate comfort and engagement."* | Hands-on, mindfulness-based OMT | |
| | / | Osteopathic active-participative approaches | |
| Baroni et al., 2021a [58] | *"Collaborative interaction based on communication and on the consensual involvement of patient and practitioner, which may result in the development of rapport, reassurance, education, shared management strategies, and patient empowerment"* | Narrative-based sense-making and decision-making process | Lunghi and Baroni, 2019 [78]; Lunghi et al., 2016 [79]; |
| | *"Touch is one of the tools to achieve a collaborative interaction with the patient, to substantiate clinical information, and to detect somatic dysfunctions: neuro-myofascial active areas that might act as an osteopath-patient interface to transmit the biological and physiological effects of touch.[...] The osteopathic touch-based examination is a collaborative interaction between osteopath and patient, that often involves palpatory feedback as an iterative process used to test hypotheses. [...] The osteopath can then more accurately determine what types of osteopathic touch-based approaches and activating forces would be most successful [...]through precise procedures and actions (such as clinical testing and examination) [...]looking for associated symptoms to pinpoint the dysfunction correlation with body functions and symptoms. [...] The impact of palpatory findings on the patient's perception (e.g., provocation tests), could act as decision making drivers, guiding the selection of both osteopathic manipulative techniques and the patient's active approaches (e.g., exercises)."* | Touch-based shared sense-making and decision-making process | |
| | *"The osteopathic palpatory specificity is not a mere motor act with standardized (or pre-determined) characteristics, but can be considered a sensorimotor transformation in which the osteopath tunes the manual input according to tissue response in a sensorimotor continuum."* | Hands-on, mindfulness-based OMT | |
| | *"Movement (used by the patient) in osteopathic approaches, micromovements, coordination and precision, pandiculation techniques, active resistant stretches, rapid stimulation and vibratory tools exercise, slow movements with myotatic reflex activation melting stretches, meditative movements, experiential bodywork, intentional breathing, intrinsic myofascial vibration exercises, low-frequency vibratory exercise."* | Osteopathic active-participative approaches | |
| Baroni et al., 2021b [59] | *"According to the complex medicine framework , there are two classes of decisions in healthcare practice, including osteopathy: decisions made by providers on behalf of patients and shared arrangements between patients and providers.[...]incorporating patient-reported outcomes, narratives, and preferences in the decision-making process may help address this problem. [...]Most decision management models in the osteopathic field are a combination of methods."* | Narrative-based sense-making and decision-making process | Sciomachen et al., 2021 [80]; Zegarra-Parodi et al., 2021[12]; |
| | *"The creation of a sense emerges from relational processes distributed in the mind-brain-body environment, where the first-person experience has a prominent role[...] Osteopathic decision-making is based on the integration of clinical history, differential diagnosis, physical examination and related multidimensional aspects (such as contextual factors, familiar symptoms, and comparable signs), allostatic load indexes, the functional assessment of self-regulation systems (B, N, RC, M, BPS), and osteopathic palpatory findings to define a tailored osteopathic treatment.[...] the focus is the patient's autonomy and interactional symmetry in the environment.[...]The palpation results are included within the mixed decision-making model* | Touch-based shared sense-making and decision-making process | |

| | | | |
|---|---|---|---|
| | *without being given a predefined value, and they are processed together with other factors to develop an individualized treatment plan."* | | |
| | *"Mind-body practice, counseling, stress management, and spirituality are integrated into the modern practice of osteopathic medicine better to address the individual.."* | Hands-on, mindfulness-based OMT | |
| | *"Osteopathic professionals also incorporate the basic principles of fascia-oriented training, archetypal postures, meditation, stress management, and mindfulness-based exercises into the patient active approach."* | Osteopathic active-participative approaches | |
| Bergna et al., 2022 [67] | / | Narrative-based sense-making and decision-making process | |
| | *"The osteopath contacts the patient's tissues by applying external forces with the hands, engaging in a relationship that leads to detecting PFs in the evaluation. The assessment of a complex system has to consider numerous variables that lead to an osteopathic diagnosis, such as clinically meaningful PFs and clinical reasoning. PFs relevant to clinical practice depend on shared decision-making and SD. Sharing the results obtained from manual evaluation with oneself, with colleagues, with other healthcare professionals, and with the patients, while maintaining its distinctiveness, should become shareable using understandable terminology and clinical outcomes.[...] Therefore, it is no longer an operator centric, it is no longer patient centric, it is a relationship centric.[...]in the shared decision-making process the patient has a part because he or she checks if what I am touching has an impact on his body functions, which he or she recognizes with his or her body awareness … the 'familiar symptoms', the patient is aware of them, he or she tells you; the 'signs of comparison' are those where the osteopath accesses through an intuition and a palpation to some aspects that are not accessible to the body awareness of the patient in that moment and therefore the verbal feedback arrives through the mediation of the palpation of the osteopath"* | Touch-based shared sense-making and decision-making process | |
| | / | Hands-on, mindfulness-based OMT | |
| | / | Osteopathic active-participative approaches | |
| Bohlen et al., 2021 [23] | / | Narrative-based sense-making and decision-making process | Lunghi et al., 2016 [79] |
| | / | Touch-based shared sense-making and decision-making process | |
| | *"Furthermore, our framework provides a theoretical grounding on which to assess multidisciplinary collaborations between mental and physical healthcare specialists to treat comorbid physical and mental symptoms or health conditions. In detail, we propose an integrative interoceptive exposure therapy that may underpin multidisciplinary person-centered care. Therein, top-down mindfulness-based psychotherapeutic interventions may be combined with bottom-up touch-based osteopathic interventions to enhance interoceptive processing and reduce physical and mental symptoms. More specifically, we propose combining modalities from psychotherapy, mindfulness, mentalization, and osteopathy to identify, attend to, perceive, and reflect on bodily (interoceptive) sensations linked to physical and mental symptoms.[...]Furthermore, recent reviews of neuroimaging studies demonstrate functional convergence for mindfulness and touch at the interoceptive cortex—this provides a rationale for investigating the combination of top-down mindfulness–informed and bottom-up touch-based approaches in the treatment of body-mind disorders that involve interoceptive deficits (), including chronic pain and depression."* | Hands-on, mindfulness-based OMT | |
| | *"Furthermore, it is currently unclear how the nature and principles of osteopathy inform patient management strategies commonly used in clinical practice; in particular, patient/pain education and psychological support, lifestyle advice emphasizing diet, nutrition, physical activity, and exercise, and self-management solutions. In summary, osteopathy arguably combines hands-on manual approaches (using touch and manipulation) informed by* | Osteopathic active-participative approaches | |

| | | | |
|---|---|---|---|
| | *osteopathic models of care with hands-off patient management approaches (using patient education, psychological support, lifestyle advice, and self-management solutions) informed by osteopathic principles, both utilizing top-down and bottom-up dynamics between peripheral tissues and the brain. Ultimately, one can argue that osteopathy may enhance the patient's knowledge and perception of health. "* | | |
| Cerritelli and Esteves, 2022 [24] | *"A robust therapeutic alliance centred on cooperative communication and shared narratives and the appropriate and effective use of touch and hands-on care enable patients to contextualize their lived experiences."* | Narrative-based sense-making and decision-making process | |
| | *"Touch and hands-on care enhance the therapeutic alliance, mental state alignment, and biobehavioural synchrony between patient and practitioner."* | Touch-based shared sense-making and decision-making process | |
| | *"Hands-on care is crucial because it effectively communicates one's cognitions and perceptions, provides context and precision, establishes an interpersonal connection, and infers another's mental states, facilitating biobehavioural synchronization"* | Hands-on, mindfulness-based OMT | |
| | / | Osteopathic active-participative approaches | |
| Cerritelli et al., 2017 [71] | / | Narrative-based sense-making and decision-making process | |
| | / | Touch-based shared sense-making and decision-making process | |
| | "[...]*using different touch-based modalities, highlighting the role of CTs in the central representation of the physical condition of the body. [...]Touch has been always regarded as a powerful communication channel playing a key role in governing our emotional wellbeing and possibly perception of self, i.e., interoceptive reactions."*[...]*osteopathic procedures that mimic the experimental study group where the operator is constantly touching the patient and contextually engaged into a focused tactile attention task, e.g., driving the attention towards the perception of myofascial movements"* | Hands-on, mindfulness-based OMT | |
| | / | Osteopathic active-participative approaches | |
| Cerritelli et al., 2020 [72] | / | Narrative-based sense-making and decision-making process | |
| | / | Touch-based shared sense-making and decision-making process | |
| | *"[…]the use of osteopathic manipulative treatment might produce specific effects in interoceptive brain areas, possibly reflecting an increased efficiency to decode bottom-up interoceptive heart-based stimuli. […] osteopathic manipulative treatment can influence activity in the rMFG allowing a more precise attentional control in order to re-orient more efficiently one's own attention towards endogenous stimuli. […] osteopathic manipulative treatment might act on the rMFG facilitating the switch from external to internal milieu. This would eventually impact on the accuracy of perception from within the body."* | Hands-on, mindfulness-based OMT | |
| | / | Osteopathic active-participative approaches | |
| Consorti et al., 2023 [60] | *"Osteopathic clinical reasoning is considered as informed by a multidimensional assessment to define person-centred treatment models. Emergent elements from patients' verbal and body narratives, standardized patient-reported outcomes measures, patient-reported experience measures, functional objective examination, and palpatory findings are implemented."* | Narrative-based sense-making and decision-making process | Liem and Lunghi, 2023 [76]; Lunghi et al., 2016 [79]; Lunghi et al., 2022 [81]; Sciomachen et al., 2018 [80]; |
| | *"This test class (i.e., Structure-Function-Correlation test) is used as a driver within a shared decision-making process and allows the operator to confirm or refute his hypotheses based on the patient's emerging responses. [...] the type of touch that is perceived as pleasant by the patient should be considered for the selection of the therapeutic approach.[...]a nonverbal exchange between the patient and the osteopath (supported by narrative* | Touch-based shared sense-making and decision-making process | |

| | | |
|---|---|---|
| | *interactions), intended to produce a pleasant surprise and a significant prediction error that will defy prior assumptions and update the brain's generative model.[...]. The presented model is consensual with the principles of person-centred care for musculoskeletal pain management. Putting person-centred care principles into practice could favour the establishment of meaningful connections by using the body as a pivot point."* | |
| | *"[...]the osteopaths select systemic maximalist approaches that involve the use of general types of touch and global body positioning to support regulatory functions (e.g., pumping type of touch to improve lymphatic flow, typically used in lymphatic pump techniques; or a type of touch which has been proposed to have an interoceptive value like fascial unwinding; mindfulness-based and meditative-ideomotor movements to balance autonomic nervous system pathway."* | Hands-on, mindfulness-based OMT |
| | *Systemic/functional approaches […] might be of use in combination with active patient approaches (i.e., mindfulness-based strategies, experiential bodywork and specific exercises).* | Osteopathic active-participative approaches |
| Elkiss and Jerome 2012 [69] | / | Narrative-based sense-making and decision-making process |
| | *"Osteopathic palpation and empathic communication allows a synchrony to occur within the patient- physician relationship. [...]What begins as a palpatory examination quickly becomes a tactile conversation as the patient gains greater proprioceptive self-awareness of structural and motion impediments causing or being caused by a dysfunction of connective tissue, vascular or neurologic structures, associated metabolic processes, or overt behaviors. With illness magnified by stress and dysregulations of the MINE systems, touch, with its unique properties, can help open the door to the patient's healing.* | Touch-based shared sense-making and decision-making process |
| | *"[...]To understand the systems-network aspect of touch, one must consider the structural and functional interactions of the musculoskeletal, immune, nervous, and endocrine (MINE) systems in response to touch. For both patient and physician, it is an intensely meaningful, heartfelt, therapeutic interaction with touch as the interface. In fact, the process of touching and talking to our patients facilitates a clinically relevant integration of psyche and soma."* | Hands-on, mindfulness-based OMT |
| | / | Osteopathic active-participative approaches |
| Esteves et al., 2022 [25] | *"Through a dyadic therapeutic relationship—a structured set of affordances—osteopath and patient actively construct a shared, sense-making narrative and realize a shared generative model of their engagement with their ecological (therapeutic) niche"* | Narrative-based sense-making and decision-making process |
| | *"Touch plays a critical role in developing a robust therapeutic alliance, mental state alignment, and biobehavioural synchrony between patient and practitioner"* | Touch-based shared sense-making and decision-making process |
| | *"Hands-on strategies in osteopathy [...]are a unique form of communication, which can be regarded as an interactive action-perception dynamic loop of the two active inference systems."* | Hands-on, mindfulness-based OMT |
| | *"As person-centred care, it is crucial that osteopaths shift away from body-centred passive models of care, to consciously consider the role of effective communication, education and reassurance—this could be conceived as psychologically informed osteopathic care."* | Osteopathic active-participative approaches |
| Groenevelt and Slatman 2024 [61] | *"[...]patients themselves will give clues as to how palpations and mobilizations are experienced: whether they are uncomfortable or painful. Taken together, these data allow the osteopath to distill whether tissue feels normal or not."* | Narrative-based sense-making and decision-making process |
| | *"[...]touch as an affective/affecting practice that allows human bodies to be configurated in particular ways."* | Touch-based shared sense-making and decision-making process |
| | *" One of the premises that underlies osteopathic treatment is that the body is hermeneutic: it requires interpretation. In interviews, osteopaths would explain how their treatment centers on finding out what bodies are trying to say[..]Touching and feeling the body is informed by various knowledge system."* | Hands-on, mindfulness-based OMT |

| | | | |
|---|---|---|---|
| | *"To facilitate optimal palpatory perception, patients are asked to undress, stand in particular poses, flex particular body parts[..] What is important to underline at this point that touching is not understood as a unilinear activity – i.e., something that osteopaths do to a patients"* | Osteopathic active-participative approaches | |
| Kim et al., 2022 [62] | *"Touch therapy is a process of establishing shared narratives through "listening to body-talk and constructing body-stories""* | Narrative-based sense-making and decision-making process | |
| | *"Touch therapy is a dynamic interactive ritual that provides opportunities for reinterpreting sensory signals, redeploying attention, and attenuating and ignoring irrelevant or ego-dystonic sensory inputs.[...]In touch with verbal guidance therapy, the therapist is supposed to simultaneously provide the sensory signals to be processed through the bottom-up route (touch) and the top-down priors to be evinced through the top-down route (verbal communication). This could make a particularly effective touch therapy, as the purpose of touch is to help the patients to learn new habits of sense-making,"* | Touch-based shared sense-making and decision-making process | |
| | *"To realign the precision or gain control mechanism of active inference, the therapist asks a series of questions directing the patient's attention to a wide range of focal points. The patients will be told that they do not have to answer the questions verbally. The patients will be encouraged to pay clear and distinct attention, or bare attention, to the mentioned parts of their bodies.[...]The therapist will provide verbal guidance and touch simultaneously; the patient will update their interpretational framework in the form of imageries, which would allow the patient to interpret the touch signals in a novel way"* | Hands-on, mindfulness-based OMT | |
| | *"[...]the strategic methods of manual touch with verbal guidance would benefit the providers of mental and physical health care services through hands-on techniques, such as osteopaths [...] According to active inference theory, the essence of touch in manual therapy is the provision of sensory data for (re)interpretation by a patient. To teach the patient a new way of interpreting interoceptive and proprioceptive signals, therapists should install a complementary set of prior beliefs—to revise generative models—through verbal communication"* | Osteopathic active-participative approaches | |
| Liem et al., 2024 [63] | *"The amplified verbal communication between therapist and participant [...] might have improved the therapeutic alliance."* | Narrative-based sense-making and decision-making process | Liem and Neuhuber 2020 [74] |
| | / | Touch-based shared sense-making and decision-making process | |
| | *"Heart-focused palpation combines osteopathic manipulative treatment approaches (i.e., touch and manipulation) with modalities from mindfulness-based interventions (i.e., changes in breathing and mental focus)"* | Hands-on, mindfulness-based OMT | |
| | *"Osteopathic manipulative treatment can be defined as a person-centred approach to healthcare that applies manual diagnosis and treatment and provides psychosocial support and advice on nutrition, exercise, and lifestyle. [...] During the treatment, [...] the participant was asked to actively perceive (but not rate) current body sensations regarding arousal, vitality, and feelings. [...] to attend to pleasant sensations from these body regions and shift the attention between regions in unison with the breathing cycles. [...] to alter their breathing rate (slow down, deepen, and attend to the breath) and to actively perceive and accept bodily sensations non-judgmentally."* | Osteopathic active-participative approaches | |
| Luchesi et al., 2022 [73] | / | Narrative-based sense-making and decision-making process | |
| | / | Touch-based shared sense-making and decision-making process | |
| | *"There is a growing number of studies with multimodal approaches in the management of these patients, combining physical and behavioral therapies such as osteopathic manipulative treatment, associating pain education and clinical hypnosis."* | Hands-on, mindfulness-based OMT | |

| | | | |
|---|---|---|---|
| | / | Osteopathic active-participative approaches | |
| Lunghi et al., 2020 [64] | *"Incorrect communication or misunderstandings regarding the type of treatment procedure may negatively influence the individual responses to treatment.[...]This includes negative messages that local biomechanical dysfunction is the sole driver behind chronic musculoskeletal disorders [...]In order to facilitate the healing process, the patient needs to give meaning to their illness experience and the healing process. The meaning itself has a therapeutic value able to trans- form the person's illness experience through placebo."* | Narrative-based sense-making and decision-making process | D'Alessandro et al., 2016 [82]; Sciomachen et al., 2018 [80]; |
| | *"The integration between the palpatory assessment and patients' perception may enable osteopaths to appraise the relevance of OPFs critically[...] Positively valenced (pleasant) exteroceptive stimuli (e.g., touch) are likely to activate the interoceptive areas of the brain and modulate pain responses. Arguably, touch-based osteopathic techniques could evoke similar responses. In contrast, negatively valenced experiences (unpleasant) may increase anxiety and pain."* | Touch-based shared sense-making and decision-making process | |
| | *"Moreover, they should also consider the role of different forms of touch-based treatment techniques and the specific physiological and behavioural effects on patient's perception [...]During the osteopathic diagnosis and treatment process, patients should be attentive to differences in pressure, mobility and tissue texture during treatment. The patient's focus and motivation should be directed away from their symptoms and their avoidance coping strategies, towards the improvement of function.[...]osteopathic manual techniques should be viewed as a vehicle to deliver touch effects, which can have a positive influence on the sense of "self", wellbeing and body image, as well as profound calming-soothing influence on the individual."* | Hands-on, mindfulness-based OMT | |
| | *"Positive effects on the "integration between interoceptive-proprioceptive-exteroceptive systems and allostasis", can be achieved primarily if an "osteopathic adaptive approach" is considered"* | Osteopathic active-participative approaches | |
| Mercadié et al., 2017 [70] | / | Narrative-based sense-making and decision-making process | |
| | / | Touch-based shared sense-making and decision-making process | |
| | *"On the one hand, these results thus confirm that music has the capacity to modulate the effect of osteopathic treatment both at the psychological and physiological level. On the other hand, it also reveals that, despite the patients' and practitioner's subjective reports, music does not amplify the effect of treatment; in fact, in our (rather unnatural) desynchronized condition, even the opposite happened: when a patient and their practitioner listened to desynchronized music, the effect of treatment (as measured indirectly by mu-ERD) was reduced."* | Hands-on, mindfulness-based OMT | |
| | / | Osteopathic active-participative approaches | |
| Vismara et al., 2022 [68] | / | Narrative-based sense-making and decision-making process | |
| | *"To evaluate the tenderness status, the reaction during slight compression between head and sacrum was evaluated using the facial expression, the presence of reflex-moves, the crying, or the sudden increase of heart rate."* | Touch-based shared sense-making and decision-making process | |
| | / | Hands-on, mindfulness-based OMT | |
| | / | Osteopathic active-participative approaches | |
| Zegarra-Parodi et al., 2023 [65] | *"[…]it is crucial that practitioners consider different patient values and expectations because they directly influence the therapeutic outcome […] practitioners should develop essential skills to perform healing rituals that combine body experience symbols with a narrative made of verbal symbols. […] As part of this inclusive approach, the Cynefin framework is proposed to help osteopathic practitioners understand culturally sensitive, patient-* | Narrative-based sense-making and decision-making process | Liem and Lunghi, 2023 [76]; Lunghi and Baroni, 2019 [78]; Lunghi et al., 2022 [81]; McParlin et al., 2022a [29]; McParlin et al., 2022b [77]; |

| | | | |
|---|---|---|---|
| | *centered care (Figure 2). The different sets of therapeutic actions and related narratives are presented in the five domains as options to help patients make sense of expected changes in their bodily perceptions according to their dominant underlying sociocultural health assumptions."* | | |
| | *"By using nonverbal behavior, proximity approaches, interoceptive touch, and mindful- based procedures that support effective communication, a better therapeutic alliance during osteopathic encounters can be created. For example, touch-based strategies are valuable for creating collaborative agreement related to goals and tasks and for the development of successful relationships and cooperative communication, especially for patients confused by sociocultural health assumptions outside their usual worldview. Touch also has a role in the development of synchrony through a more precise categorization of individuals, where more adaptive feedback loops are created to minimize surprise, increase understanding, and reduce physical and psychological stress, all of which are crucial for daily living."* | Touch-based shared sense-making and decision-making process | |
| | *[…] For example, passive manual approaches […]may be proposed and combined with active approaches, such as lifestyle counselling, exercise, and nutritional advice, and with top-down strategies, such as mindfulness for stress management. Further, person-centered osteopathic care that includes the spiritual dimension in healthcare can be proposed as another top-down strategy when using this framework.[…]* | Hands-on, mindfulness-based OMT | |
| | *[…] For example, passive manual approaches […]may be proposed and combined with active approaches, such as lifestyle counselling, exercise, and nutritional advice, and with top-down strategies, such as mindfulness for stress management. Further, person-centered osteopathic care that includes the spiritual dimension in healthcare can be proposed as another top-down strategy when using this framework.[…]* | Osteopathic active-participative approaches | |
| Zegarra-Parodi et al., 2024 [66] | *"The implementation of the Cynefin Framework in sense-making processes and clinical reasoning […] help both patients and practitioners hold a holistic view and to consider the role of psychological and existential domains in health while simultaneously applying biological and biomedical aspects to make shared clinical decisions. […] a safe contemplative space, allowing patients to self-reflect on difficult situations, remain suitably undecided, and meditate. This facilitates connecting with potential solutions that patients feel are viable, thereby initiating their integration into the context and environment. […]Owing to the negotiation process facilitated by the practitioner, the patient was invited to reformulate the confusing elements of his health. Consequently, the patient could reformulate confused things, shifting them to other domains (i.e., simple, complicated, complex, and chaotic), and applying different reasoning. "* | Narrative-based sense-making and decision-making process | Barsotti, et al., 2023 [83]; Baroni et al., 2023 [84]; D'Alessandro et al., 2016 [82]; Zegarra-Parodi et al., 2021[12]; |
| | *"A culturally sensitive narrative, following a neuroaesthetic–enactive experience, introduces how their physical body might influence the complex interaction between body systems, i.e., how musculoskeletal function amenable to manual approaches might impact biomechanical, neurological, respiratory–circulatory, metabolic–energetic, and behavioral or biopsychosocial processes involved in individual health. […] Osteopathic practitioners suggest a minimalist approach according to body areas of interest considered clinically relevant by both the patient and the practitioner following manual and functional objective examination, with the term 'somatic dysfunction' coined to represent a patient-emergent pattern showing a relation between body functioning, patient ability to perform daily activities, and elements of the body framework."* | Touch-based shared sense-making and decision-making process | |
| | *"Osteopathic practitioners should use maximalist approaches, such as interoceptive touch-based mindfulness strategies, close operator–patient proximity, and nonverbal behaviors, to evoke patient patterns and enhance psychological flexibility and self-regulation functioning."* | Hands-on, mindfulness-based OMT | |
| | *"Assisted exercise, lifestyle education, empathic communication strategies, and behavioral approaches, based on practitioner–patient proximity and non-verbal behaviors are integrated with minimalist and maximalist manipulative methods [...]Mindfulness-based strategy (i.e., body scan and intentional breathing) and experiential bodyworks (e.g., functional neuromyofascial activity) integrated with osteopathic manipulative approaches (e.g., integrated neuromusculoskeletal release), background* | Osteopathic active-participative approaches | |

*music during osteopathic treatment, the use of essential oils during hands-on treatment and self-care strategies."*
